# Patient and family organization perspectives on poor treatment in Swedish adult psychiatric care

**DOI:** 10.1007/s44192-026-00372-0

**Published:** 2026-01-22

**Authors:** Ellen Gustafsson Lindh, Maja Magnusson Skog, Henrik Levinsson, Martin Wolgast

**Affiliations:** Department of Psychology, Lund University, Box 213, 221 00 Lund, Sweden

**Keywords:** Psychiatric care, Patient experiences, Epistemic injustice, Respect and integrity

## Abstract

Respect and integrity are fundamental to effective psychiatric care, yet patient experiences of poor treatment remain a significant concern. This study explores patient perspectives on mistreatment within Swedish adult psychiatry through focus group interviews with representatives of patient and family organizations. Participants described systemic and interpersonal challenges that contribute to inadequate care, identifying four main themes: inaccessibility of psychiatric services, lack of collaboration between patients and healthcare providers, absence of a holistic perspective on patients, and a stark power imbalance between vulnerable individuals and an authoritative psychiatric system. Structural barriers, including long wait times and limited treatment options, were perceived as forms of neglect, leaving patients struggling to access necessary care. Patients frequently felt excluded from decision-making, as their lived experiences were often dismissed in favor of standardized treatment protocols. This lack of recognition was further compounded by epistemic injustice, where patients' accounts were deemed unreliable due to prevailing biases against psychiatric populations. Participants emphasized the emotional toll of dismissive encounters, with many patients reporting feelings of helplessness, mistrust, and self-doubt. The study highlights the urgent need for reforms that prioritize respect, collaboration, and patient-centered care in psychiatric settings. Addressing these concerns requires systemic changes to reduce barriers to care, improve communication, and ensure that psychiatric patients are treated as credible, autonomous individuals. By acknowledging patient perspectives, psychiatric care can foster greater trust, improve adherence to treatment, and ultimately enhance mental health outcomes.

**Supplementary Information:**

The online version contains supplementary material available at 10.1007/s44192-026-00372-0.

## Introduction

Respectful treatment is associated with better outcomes, including higher satisfaction and adherence [5], whereas poor interactions can contribute to delayed help-seeking, strained patient–provider relationships, and lower care quality [31]. Such experiences may also reduce trust in healthcare, as shown by 27.8% of Norwegians reporting low trust in psychiatric care [29]. In Swedish adult psychiatry, 96% of staff have witnessed ethical violations and 84% have committed them [33], yet little is known about how patients themselves experience poor treatment. This study therefore explores these issues through focus group interviews with representatives of patient and family organizations.

The term “poor treatment” refers to subjective experiences of insufficient care that diminish dignity and self-worth [8]. Related concepts include dignity violations, ethical transgressions, and abuse [27, 33]. Here, the term is defined broadly to capture interactions perceived as unsatisfactory, regardless of whether they meet formal criteria for violations.

Psychiatry encompasses multiple professions and differing understandings of mental illness. Debates concern the causes of mental disorders, the role of diagnoses, and how to approach comorbidity [11]. While psychiatry has tended toward a medical model, tensions remain between biological, psychological, and social explanations [16, 37]. Such divergences may contribute to mismatches between patient and provider perspectives, influencing experiences of treatment quality.

## Power and epistemic injustice in healthcare settings

Power asymmetries are inherent in healthcare, where professionals hold authority over knowledge and access to treatment, including involuntary care [22]. These imbalances can reduce provider effort and negatively affect outcomes [40], while patients who experience powerlessness report heightened stress and negative emotions [46].

Epistemic injustice [21] further shapes interactions. Testimonial injustice arises when patients are deemed less credible due to stereotypes,hermeneutical injustice occurs when individuals lack the conceptual resources to articulate their experiences. In psychiatry, these dynamics may manifest when patients’ accounts are dismissed as unreliable because of assumptions regarding cognitive impairment or emotional instability [10]. Psychiatric patients are particularly vulnerable due to pervasive stigma [13].

Autonomy is intended to counteract power asymmetries by supporting patient agency. However, autonomy without relational support can leave patients isolated in decision-making. Relational autonomy emphasizes that supportive interactions enhance agency [18].

## Stereotypes and stigma

Stigma marks individuals as deviant and less valued (Public Health Agency of Sweden 2020) and contributes to prejudice and self-stigma [12]. Although stigma can deter help-seeking, belief in treatment effectiveness mitigates these effects [12]. Provider attitudes significantly shape stigma,pessimism, low competence, and unacknowledged biases contribute to discriminatory practices [31]. Nurses, for example, may more often view individuals with substance use disorders or schizophrenia as dangerous, particularly in somatic settings [6].

## Psychiatry as a workplace

Workplace conditions also affect patient treatment. Compassion fatigue—a combination of burnout and secondary traumatic stress—can impair concentration, reduce empathy, and negatively impact care [19, 42]. High workloads and limited support increase these risks, as seen in studies of psychologists [30]. To cope, staff may dehumanize patients, particularly those perceived as low in warmth and competence [23], reducing empathy and willingness to help [3]. Infrahumanization further prioritizes in-group needs [38]. Dehumanization has been found to be more common toward psychiatric than somatic patients, but decreases when staff experience positive interactions and increases when they feel dehumanized by superiors [20].

## Risk factors and prevalence for poor treatment

Certain groups are more likely to report poor treatment, including women, younger individuals, and those with symptoms difficult to verify [28]. Previous experiences of abuse increase vulnerability in healthcare encounters [45]. Dignity violations often occur when patient needs intersect with resource constraints or routine procedures [27], and are influenced by patients’ ability to advocate for themselves [9].

Because definitions of poor treatment vary, prevalence estimates differ across studies. Research has explored abuse in healthcare [8, 43], ethical violations [33, 44], and other forms of mistreatment [26]. In Swedish general healthcare, 73% of female patients report ethical violations [44], and 38% of the population reports poor treatment, with 10% reporting repeated incidents [28]. In psychiatric settings, discrimination and dismissive treatment are commonly reported internationally [1, 26]. Swedish data similarly show widespread ethically problematic behaviors in adult psychiatry [33].

Qualitative studies identify valued components of care—clear communication, predictability, competence, availability, individualized care, and secure relationships [4, 35]—and describe poor treatment as involving violations of autonomy, lack of empathy, dismissiveness, indifference, overprotectiveness, low competence, and withholding of information [24, 25, 46].

## Purpose and research questions

It is evident that various forms of ethical violations and mistreatment occur in healthcare generally. Numerous studies have examined patients’ experiences of poor treatment in somatic care. However, similar research focusing on Swedish adult psychiatry is lacking. Patient and family organizations in Sweden possess a broad and comprehensive understanding of the experiences of psychiatric patients.

The aim of this study, therefore, is to provide a broad patient perspective on poor treatment in Swedish adult psychiatry. This will be achieved through focus group interviews with representatives from patient and family organizations. The specific research question addressed by the study is:

*"What perspective do representatives of patient and family organizations have on poor treatment in Swedish adult psychiatry, based on the experiences of their members?"*

## Methods

A critical realist approach was adopted to understand experiences of poor treatment in Swedish adult psychiatry, recognizing that the data reflects participants’ interpretations and is further shaped by the researchers’ analysis.

Focus groups were conducted with representatives from patient and family organizations, which provide support and advocate for improvements in psychiatry. These organizations, some of which have produced reports on treatment (e.g., [32, 41]), were selected for their collective understanding of poor treatment based on member experiences.

Focus groups were chosen for their conversational format, allowing participants to direct discussions, highlight priorities, and build on each other’s ideas, providing both individual and collective perspectives [14].

Data was analyzed using Braun and Clarke’s reflexive thematic analysis [7], suitable for identifying themes and capturing patient perspectives. Reflexive work was integral, as the researchers approached the data from a caregiver perspective. An inductive approach allowed participants’ views to be expressed naturally, avoiding the imposition of predefined frameworks like ethical guidelines.

### Participants

The study recruited representatives from patient and family organizations. These organizations provide direct support to individuals and families, advocate for patient rights, and influence public debate and policy. Several have also produced independent reports documenting treatment deficiencies and proposing reforms. By recruiting representatives from such organizations, the study accessed collective perspectives informed by the lived experiences of their members. This approach provided a broad overview of common concerns across psychiatric care settings, rather than individual narratives alone.

Inclusion criteria required participants to represent organizations for individuals interacting with Swedish adult psychiatry, speak Swedish, and be over 18 years old. Recruitment occurred between January and February 2024. Relevant organizations were identified, including those for individuals frequently co-diagnosed with psychiatric conditions, such as neurodevelopmental disorders and substance dependence. Fourteen organizations were contacted via email, which included study information and a request to share the invitation with their representatives. Eleven representatives expressed interest. One was excluded for not meeting criteria, one withdrew, one was unable to attend due to illness, and one did not show up. Thus, seven participants joined the focus groups, representing various regions of Sweden and patient groups, including neurodevelopmental disorders, eating disorders, affective disorders, and immune-psychiatric conditions. Broader, umbrella organizations were also included. Participants’ organizational experience ranged from 2 to over 10 years.

Seven representatives from patient and family organizations participated in the study. Among them were individuals with personal lived experience, family members/carers, and organisational advocates with long-standing involvement in psychiatric patient issues. To protect participants’ anonymity, we chose not to present potentially identifying demographic information (e.g., gender, organizational type, years of experience, or region) and we did not attribute quotes to individual participants. Because participants represented national and regional patient and family organizations, even limited identifiers could risk recognition by insiders familiar with the organizational context. Instead, we emphasize the collective perspectives that were identified across organizations. This decision reflects a balance between transparency in reporting and our ethical obligation to safeguard confidentiality.

### Data generation

Two focus groups were conducted digitally via Zoom in January and February 2024, lasting approximately 2 h each with a short break. Four participants attended the first group and three attended the second. Both sessions were facilitated by the first and second authors (EGL and MMS), who are female final-year psychology students with prior training in qualitative methods. Their perspective as clinicians-in-training informed their facilitation style, which emphasized openness, reflexivity, and participant-led dialogue. At the beginning of each session, participants were informed about the authors’ motivation to contribute to improved psychiatric care. No prior relationship existed between researchers and participants. Discussions followed a semi-structured interview guide (see Supplementary material), and were audio-recorded in Zoom. Field notes were taken during and after each session. The first and second authors transcribed one session each and reviewed each other’s transcripts for accuracy. Beside the researchers and the participants, no one else was present at the interviews.

### Data analysis

Data were analyzed using Braun and Clarke’s [7] reflexive thematic analysis, supported by Nvivo software (version 15.1). Coding was conducted independently by the first and second authors, who then collaborated in refining and combining codes into preliminary themes. The full research team contributed to iterative reviews of theme structure and interpretation. Reflexivity was integrated throughout the analytic process: the first and second authors, as psychology students and future clinicians, explicitly reflected on how their caregiver perspective might shape interpretation. The senior authors contributed additional perspectives as experienced researchers and psychologists. Sample adequacy was evaluated using the concept of information power [34], rather than saturation. Given the breadth of organizations represented, the focused aim of the study, and the depth of discussion in each focus group, the seven participants were assessed as sufficient to address the research question.

## Researcher reflexivity and positionality

Reflexivity was an integral part of the study design and analysis. The first and second authors (EGL and MMS) are female final-year psychology students who, at the time of the study, were training as clinicians. Their educational background and emerging professional identities meant they approached the research with a dual perspective: as caregivers-in-training familiar with psychiatric settings, and as researchers seeking to critically examine these settings. The senior authors (HL and MW) are experienced researchers and one ow them (MW) is a licensed psychologists with longstanding interest in ethical issues in psychiatry. The researchers’ positions inevitably shaped the data collection and interpretation. While the facilitators sought to adopt an open, non-directive stance, they were also aware that their background in psychology could influence both the questions asked and the weight given to particular themes. To address this, the team engaged in continuous reflexive discussions and documented analytic decisions throughout the process. This reflexivity supported transparency and helped ensure that the voices of participants remained central in the analysis.

### Ethics

The study was approved by the Swedish Ethical Review Authority (reference number: 2023-07197-01). Participants received a consent form outlining the study’s purpose, methodology, potential risks, data handling, and their right to withdraw at any time. In this study, participants were not asked about their personal experiences, though representatives of patient and family organizations may have had such experiences. They could submit questions in writing or request a call for clarification. At the start of focus groups, participants were reminded of this information.

## Results

Based on the research question, *"What perspective do representatives of patient and family organizations have on poor treatment in Swedish adult psychiatry, based on their members’ experiences?"* four main themes were generated, two of which include sub-themes. The four main themes were: (1) *Psychiatry is Inaccessible*; (2) *There Is No Collaboration* (with the subthemes *Psychiatry Overlooks an Essential Resource: The Patient* and *A Passive Psychiatry*), (3) *A Lack of Holistic Perspective on the Person*; (4) *A Vulnerable Individual Meets a Powerful System* (with the subthemes *Healthcare Providers Do Not Hear the Patient* and *An Encounter That Sows Doubt*).

**Fig. 1:**
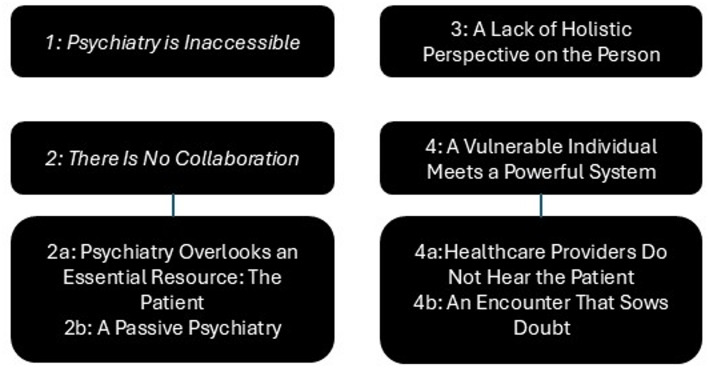
Themes and subthemes of shortcomings in patient encounters in Swedish adult psychiatry

Together, these themes provide a comprehensive view of how patient and family organization representatives perceive poor treatment in Swedish adult psychiatry, reflecting both systemic and interpersonal challenges. The themes and subthemes are presented in Fig. 1, and are extensively described below.

### Theme 1: psychiatry is inaccessible

Participants consistently described systemic barriers to accessing psychiatric care, focusing on challenges in initiating contact and maintaining access. Difficulties included long waiting lists, entry systems mismatched with patient needs, and high care thresholds, leaving many feeling poorly treated. Some noted patients were denied care for not appearing “sufficiently” unwell. One participant observed, *“I have seen more and more people fall through the cracks because they are on the borderline”.* Decisions often prioritized somatic urgency over subjective distress:

*"If someone is perceived to have restrictive eating disorders and is somatically failing [...] then it is ‘more acute,’ even if that person paradoxically feels better and may not want help at that time."*

High care thresholds also led patients to exaggerate symptoms to receive treatment*: “‘Okay, I’ll try saying I’m suicidal,’ and then they might get help, even hospitalization, even though they don’t actually have suicidal thoughts.”*

Even after contact was established, patients continued to face difficulties securing or retaining care, especially those with rare conditions. Satisfaction increased with specialist access, but the process was often lengthy and confusing. One participant noted, *“Even the staff don’t know where to refer people.”* Treatment inaccessibility extended to modalities, with many patients wanting therapy but only receiving medication. Short consultations and inconsistent providers worsened frustrations. One shared, *“After 20 min, they say, ‘Your time is up.’ You’re left standing there, having just started to open up.”* Repeatedly recounting traumatic histories to new doctors hindered progress: *“You focus on the illness instead of looking forward to solutions. It feels like going nowhere.”*

Participants emphasized these organizational issues stemmed from systemic resource constraints rather than individual negligence, yet they resulted in dismissive care. Structural barriers disproportionately affected those with limited resilience, making minor obstacles overwhelming. As one explained, *“I’ve heard things like, ‘I went to the waiting room, but something happened, and they didn’t see me, so I left because I just couldn’t handle it.’”.*

Resource shortages impacted both patients and staff, discouraging professionals from working in psychiatry. One noted, *“People don’t want to work in psychiatry anymore because their concerns aren’t heard, no matter how much they complain. Managers don’t listen, and it’s all tied to resources.”* However, some argued staff attitudes contributed, noting differences between general hospital and psychiatric care. A participant remarked, *“If there’s anywhere you should be met with understanding, it’s psychiatry.”*

Participants also questioned separating systemic issues from individual responsibility: *“We are the system. Together, we create this.”* This underscores that collective actions shape systemic failures.

Inaccessibility were expressed as a form of inadequate care, signaling to patients they must prove their need and fight to retain access. The spontaneous mention of inaccessibility in discussions of poor treatment highlights its centrality as a barrier, creating an environment that feels unwelcoming and dismissive.

### Theme 2: There is no collaboration

Participants highlighted how patients often feel a lack of collaboration with healthcare providers. Two subthemes were identified: *Psychiatry overlooks an essential resource: the patient* emphasizes the exclusion of patients and families from care decisions, while *A passive psychiatry* describes how patients are left with excessive responsibility for their own care without sufficient support. One participant remarked, *“[…] there’s no sense of ‘we’ in the care. Patients don’t feel like they’re part of a team with the doctors or psychologists or whoever it is, but instead, it’s ‘me’ and ‘them.’”* This illustrates the absence of a collaborative dynamic in care.

#### Psychiatry overlooks an essential resource: the patient

Several participants stressed the importance of valuing patients as resources in their care. Patients bring crucial insights about their conditions, yet psychiatry often neglects this expertise.

1: *“I completely agree. Patients need to feel like a valued resource, that what they say matters to those working with them. That alone is a step toward recovery. […] Instead of wasting money on things that are ineffective and downright foolish, why not ask the patients themselves, ‘What can we do?’”*

2: *“Even just experiencing that they truly listen to you and believe you, that they want to know what you think and what you need.”*

3: *“Yes, that’s something often forgotten, regardless of healthcare setting: ‘What do you need?’ Ask that question. You’ll get an answer—from the patient.”*

Participants voiced frustration at psychiatry’s failure to involve patients in care planning and evaluation. This neglect is compounded when patients’ efforts to engage are dismissed:

*“They feel that doctors are arrogant, particularly with [less common conditions], where there isn’t much knowledge yet. When family members or the patient themselves, who might feel relatively well, read up on their condition or gain experience-based knowledge over the years, it’s not received positively. Instead, we often hear that both patients and families are accused, as if it’s a bad thing, with comments like, ‘You’ve been reading up online’ or ‘We know better than you.’”*

Participants noted family members could offer valuable insights, yet their involvement is often dismissed: *“If someone around the patient shows interest, we should reciprocate that rather than immediately bringing up confidentiality before understanding their intentions.”*

However, some acknowledged family involvement could be complex, as certain dynamics might be “destructive.” Yet participants found it odd when staff discouraged patients from including family: *“There are patients who have experienced staff saying things like, ‘You’re an adult; you shouldn’t need someone with you.’”* further excluding patients from decisions about their care.

#### A passive psychiatry

Participants emphasized that collaboration requires active involvement, yet patients often face minimal engagement from providers.

*"You hear that they meet their psychiatrist once a year. It’s very difficult—they call to get an appointment, wait four weeks, and then the doctor is sick, and no one calls to reschedule. There’s a lot of this kind of thing."*

This illustrates how responsibility to reschedule often falls on patients, adding to their burden.

Patients frequently reported struggling to navigate a bureaucratic system without adequate guidance: *“We want an autism coordinator to manage contacts and provide support […] it’s hard to keep track of all the contacts and know what you’re entitled to.”* Without consistent involvement, patients feel abandoned. *“Yes, our group sees the same thing. There’s so little knowledge still, so patients are shuffled around or just left year after year, feeling forgotten.”*. This lack of support also places significant stress on families: *"[Patients’ relatives] take on a significant burden, and I think they often end up on sick leave themselves, feeling unwell because their sick adult children haven’t received help. They worry, ‘What happens when I’m old? Who will take care of my son who’s on the streets and can’t get help?’”.*

Finally, psychiatry’s passivity often shifts responsibility onto patients, expecting them to articulate needs without guidance: *"It can be good or bad to be misunderstood and mistrusted, and it can be extremely bad to be trusted if you haven’t chosen the right puzzle pieces or words. I’ve seen this too. Because then you can be mistreated for something you don’t have."*

By relying on patients to perfectly articulate their conditions, providers fail to fulfill their role as active collaborators, leaving patients confused and unsupported.

### Theme 3: a lack of holistic perspective on the person

Participants highlighted a disconnect between psychiatry’s approach and patients’ holistic needs. Patients often encounter a system that focuses on isolated parts rather than treating them as integrated individuals.

*"I sometimes wonder, how can they divide things up like that? It’s funny, someone said, ‘Going to the doctor is like going to the meat counter at the grocery store. There’s the cutlet here, something else there, and another thing over there. That’s how they see the human body, too. But where’s the soul?’”*

This analogy reflects patients’ feelings of fragmentation, where treatment focuses on specific issues rather than addressing the whole person. Such a narrow approach often limits treatment options, as providers favor pre-determined protocols over alternative interventions.

Participants noted that evidence-based guidelines sometimes hinder individualized care. *“It’s strange because those who provide the best care and treatment often don’t follow the standard regulations. […] They’re forced to break the rules to provide patient-centered care.”*

A focus on diagnoses was a recurring concern, as it often neglected the broader context of patients’ lives. One participant noted:

*"If someone grows up in a home with constant drinking, fighting, and sexual abuse, and later gets a diagnosis—how interesting is that diagnosis, really? The important thing is what they’ve been through that caused what we call a diagnosis. [...] What trauma are they carrying? That’s where we should focus."*

This tension between psychiatry’s symptom-based focus and patients’ lived experiences reduces patients to diagnostic labels, often overlooking complexities like comorbidities. *“Most of the patients we meet don’t seem to behave like those patients [described in evidence-based protocols].”*

Participants felt the rigidity of protocols left patients struggling to fit within predefined frameworks, making them feel dismissed and misunderstood.

Acknowledging psychiatry’s limitations, participants emphasized the value of genuine efforts to empathize with patients:

*"We’ve talked about the importance of user experience, because you can’t, unfortunately, learn this in books. Sorry, students, but I mean no harm—you just can’t fully understand unless you’ve experienced it yourself. But that doesn’t mean you can’t show understanding and take an interest in another person and what they’re saying."*

The disconnect between psychiatry’s frameworks and patients’ realities underscored a need for a shift toward empathy and flexibility, prioritizing individual narratives over rigid protocols. Participants argued that such changes are essential for meaningful and effective care.

### Theme 4: a vulnerable individual meets a powerful system

Participants described how patients seeking psychiatric help often face dismissive attitudes, especially in critical moments, which can discourage further help-seeking. This theme is explored in two subthemes: *Healthcare Providers Don’t Hear the Patient* and *An Encounter That Sows Doubt.*

#### Healthcare providers don’t hear the patient

Participants shared that patients frequently feel dismissed, questioned, or unheard in their interactions with psychiatry. Many described patients’ statements being ignored or contradicted by medical records that didn’t reflect their experiences.

In some cases, patients struggled to communicate their suffering in ways staff could understand. One participant explained this as, *“[The patient] speaks autistic, [the staff] speak neurotypical.”* Another noted that unclear communication often led to patients in acute distress being sent home: *“Every time the system has failed people seeking help, it’s been, ‘No, but we believe you can make it to Monday.’”.*

Beyond communication difficulties, some staff outright rejected patients’ accounts:

*“[...] the hard part is not being believed, and many testify to this. That what they say is questioned, as though the person across from them has the right to challenge what I know about myself.”*

Participants highlighted how dismissal is particularly stark when compared to somatic healthcare:

*They don’t feel taken seriously and even feel blamed. […] When you go to a hospital with something like strep throat, they look at your throat and give you antibiotics, or if it’s more serious, they admit you and give you the care you need—you’re not questioned like this. […].*

In inpatient or involuntary care settings, this sense of dismissal can be even more pronounced. One participant recounted: *“They feel very alone, saying, ‘I saw two staff members and called out to them, but they didn’t come.’”.*

#### An encounter that sows doubt

Participants emphasized how dismissive treatment in psychiatry impacts not only access to care but also patients’ confidence in recovery. Poor treatment can diminish self-worth, with one participant explaining what patients might feel: *“‘What’s wrong with me? Why can’t I get help like everyone else?’”.*

This guilt and self-doubt are sometimes reinforced by psychiatric staff, who attribute lack of improvement to patients themselves:

*“Over time, if they haven’t received the right treatment or response, it’s eventually seen as something to do with the person’s inherent abilities or insights [...]”*

These experiences can lead patients to stop seeking help entirely:

*“Many people I talk to end up stopping their care-seeking entirely because it’s pointless—they don’t get what they need anyway. They’re gaslighted into thinking, ‘Well, this is just how it’s supposed to be, nothing’s wrong, I’m not unwell, this is normal for everyone.’ And so, they don’t reach out because it’s too exhausting.”*

Participants noted how this dynamic undermines patients’ ability to advocate for themselves:

*“They don’t stand up for their rights, don’t report mistakes, even though they should. If you’re sensitive or vulnerable to this, you can become conditioned to let go of what’s your right.”*

Dismissive treatment also affects family members, influencing their ability to support the patient:

*“The way patients are treated can affect everyone around them. For example, if they’re not believed, it can carry over to family members who stop believing them and reduce their efforts to help.”*

Participants highlighted the severe consequences of poor treatment, including hopelessness and suicidal ideation:

*“If they’re treated poorly, then it’s like, ‘Not even healthcare can help me. So, I might as well end my life.’”*

Through the subthemes *Healthcare Providers Don’t Hear the Patient* and *An Encounter That Sows Doubt,* participants described vulnerable patients whose confidence is undermined by dismissive treatment. These encounters reveal psychiatry’s significant responsibility to ensure patients feel heard and supported.

## Discussion

This study contributes to the limited research on patients’ perspectives of poor treatment in Swedish adult psychiatry by highlighting systemic and interpersonal challenges described by representatives of patient and family organizations. The four themes identified -inaccessibility, lack of collaboration, absence of a holistic perspective, and power imbalance—resonate with previous research on ethical violations and mistreatment in healthcare [26, 33]. Together, they illustrate how disrespectful treatment can be embedded both in the organization of psychiatric services and in the everyday encounters between patients and staff.

### Psychiatry as a system

Participants emphasized the challenges of accessing psychiatry, noting that even when patients gain access, adequate care often remains elusive. Similar to Ashcroft et al. [4], accessibility was highlighted as critical, with patients struggling to obtain psychotherapy and long-term care, often receiving only medication. While medication may relieve symptoms, participants stressed it does not address deeper issues. This critique reflects concerns that psychiatry’s medical model prioritizes biological perspectives at the expense of psychological and social ones [16, 41].

Resource shortages were frequently cited as a cause of inaccessibility, consistent with national reports of long wait times [51]. While staff often have good intentions, systemic constraints limit their ability to act, contributing to empathy fatigue [30]. Reduced empathy has been linked to dehumanization in patient care [3], echoing Husum et al.’s [26] finding that lack of empathy is a central problem in psychiatry. These systemic failures thus harm both patients and staff, perpetuating inadequate care.

### Balancing systemic and individual accountability

The inaccessibility of psychiatry, while largely systemic, also manifests in individual encounters. Participants noted that staff are part of the system but also agents within it, and therefore share responsibility for its functioning. Some warned against overemphasizing structural issues, as this risks obscuring caregiver accountability. While many participants avoided blaming staff for systemic limitations, others argued not all shortcomings can be attributed to systemic constraints alone. This highlights the need to address both organizational and interpersonal aspects of care to achieve change.

### Autonomy, care, or collaboration

A key finding was the lack of collaboration between patients and healthcare providers. Participants felt excluded from decisions and left to manage their treatment alone. This aligns with research on psychiatry dismissing patients’ input [25] and withholding information [46]. The subtheme *Psychiatry Overlooks an Essential Resource: The Patient* illustrates how patients’ attempts to engage are often dismissed. Similar patterns have been reported in somatic care, where contributing knowledge can paradoxically result in inadequate treatment [9].

The overarching theme *There is No Collaboration* shows how both excessive provider control and insufficient support undermine care. This reflects relational autonomy [18], in which autonomy depends on meaningful collaboration rather than either control or neglect. Dismissing patients as sources of knowledge also exemplifies epistemic injustice [21], reducing agency and depriving care of valuable insights.

### Psychiatry’s authority and patient powerlessness

Participants described inadequate communication in psychiatry, including misunderstandings and dismissiveness by staff. These issues are captured in the theme *A Vulnerable Individual Meets a Powerful System*. Failures in communication and questioning patients’ experiences led to self-doubt and a loss of voice, echoing broader critiques of power imbalances in healthcare [22].

One participant emphasized disparities between somatic and psychiatric care, noting that patients are taken more seriously in somatic contexts. This may reflect epistemic injustice, where credibility depends on perceived identity and psychiatric symptoms undermine trust [13]. Participants highlighted that, while somatic care often relies on objective tests, psychiatry demands greater reliance on subjective reports, which may intensify dismissive attitudes. Regardless of objectivity, the findings show patients perceive significant differences in how they are treated in psychiatric versus somatic contexts.

### Consequences of poor treatment

Participants also discussed the profound consequences of poor treatment in psychiatry, including a loss of trust in both the system and themselves, encapsulated in the subtheme *An Encounter That Sows Doubt.* Similar findings have been reported elsewhere, where low trust in psychiatry diminishes patients’ likelihood of seeking help [29]. Trust in effective treatment is a critical factor in overcoming barriers to care [12] and is particularly important in psychotherapy, where positive expectations strongly influence outcomes [47]. Thus, fostering trust in psychiatric care is essential for improving patient experiences and outcomes.

The theme *A Lack of a Holistic Perspective on the Person* highlights how patients feel disconnected from a healthcare system that prioritizes diagnoses, symptoms, and standardized processes over their complex self-identity. Participants expressed a desire to be seen as whole individuals rather than fragmented components. This aligns with research emphasizing the need to address diverse patient needs [4]. While prior studies often focus on cultural differences, this study emphasizes recognizing the individual as a complete person rather than a collection of symptoms. Similar tensions have been observed in somatic care, where rigid frameworks can leave patients feeling inadequately treated [27]. Symptoms that are hard to verify, such as mental health issues or fatigue, increase the risk of perceived mistreatment [28]. This reflects a broader tendency to prioritize objective findings and expertise over patients’ subjective experiences.

### Criticism of standardized approaches

Participants criticized manualized treatments for failing to accommodate the realities of individual patients. Similar concerns have been raised by clinicians, who note that treatment manuals often presuppose ideal conditions rarely present in practice [2]. The clash between patients’ personal narratives and diagnostic frameworks can be understood as hermeneutical injustice [21]. Overemphasis on diagnoses may also reinforce stigma and stereotypes [39], further marginalizing individuals and reducing empathy among providers [3].

### Humanizing the patient experience

Several participants stressed that care quality depends not only on diagnoses or treatment plans, but on how these are conveyed. Patients valued communication that acknowledged the limitations of standard treatments while framing them as steps within a broader understanding of the individual. This suggests that shortcomings may extend beyond negative effects of the use of diagnostic frameworks to a more general absence of a humanistic, collaborative outlook. Research supports that dehumanization decreases when staff–patient rapport is strong [20], underscoring the importance of empathy and transparency in psychiatric encounters.

### A holistic perspective on shortcomings in patient encounters

The four themes identified in this study are interconnected and overlap significantly, reflecting participants’ holistic perspective on shortcomings in psychiatric care. Rather than isolating specific behaviors in individual situations, participants described a broader framework where shortcomings stem from a lack of comprehensive, person-centered care. This interpretation is reinforced by the consistency of responses across diverse participants, who represented patient and family organizations from different regions and groups. Despite some situations linked to specific diagnoses, most issues described were seen as universally human.

In healthcare guidelines, there is often a balance between general principles (e.g., “treat the individual with respect, empathy, and attentiveness” [50]) and specific behavioral instructions (e.g., "summarize," "make comfortable eye contact," "maintain open body language" [50]). Participants indicated that effective patient care may require more than behavioral checklists, calling instead for a foundational humanistic outlook. Several participants emphasized that genuine understanding arises from personal experience or a sincere interest in the patient’s perspective, framing good encounters as acts of shared humanity.

An additional strategy that has been highlighted in international research to counteract neglect of person-centered care is the integration of peer support workers—individuals with lived experience of mental illness—into psychiatric treatment teams. Peer involvement can help reduce the power asymmetry between professionals and patients, foster greater empathy, and promote collaboration [15, 36]. Although this approach was not explicitly raised in our focus groups, the themes identified in our study, particularly those concerning lack of collaboration and dismissal of patient perspectives, suggest that peer support may represent a valuable avenue for reform in Swedish psychiatry. Future research could examine how peer involvement might address some of the systemic shortcomings described by participants.

### Strengths and limitations

This study’s strengths and limitations are discussed using Elliott et al.’s [17] quality criteria for qualitative research. Two key aspects emphasized by Elliott et al. are the clarity and coherence of results and the transparent connection between findings and data. Efforts were made to clearly outline how interpretations were derived from the data, allowing readers to assess the robustness of the identified themes. For example, while some statements could fit under multiple subthemes, such as *Healthcare Providers Don’t Hear the Patient* and *Psychiatry Misses an Important Resource: The Patient,* we carefully distinguished between them to ensure clarity.

Elliott et al.’s sixth criterion highlights the importance of aligning methods with research aims. This study aimed to capture a broad understanding of patient perspectives on shortcomings in Swedish adult psychiatry, adopting a general research goal [17]. While the relatively small number of participants is a limitation, the inclusion of representatives speaking on behalf of their members increased the breadth of perspectives. Participants represented diverse regions and patient groups, enhancing transferability across contexts. However, the focus group format, while facilitating agreement and interaction, may have limited the diversity of topics discussed compared to individual interviews.

The choice to interview representatives allowed for a synthesized view of patient experiences but also introduced interpretative layers. These findings should be understood as reflections of how representatives perceive their members’ experiences rather than direct patient accounts. Moreover, our professional perspectives and interests inevitably influenced our interpretations, aligning with Elliott et al.’s call to disclose researcher bias. While we sought to address this reflexively, unacknowledged biases may remain.

Another limitation relates to the theoretical framework and the definition of "shortcomings in patient encounters," which lacks an established English equivalent or clear definition. This necessitated a careful delineation of the concept’s aspects, as it encompasses systemic inaccessibility and interpersonal dismissiveness.

#### Recommendations and future research

Previous research indicates that ethical breaches in psychiatry are widespread, with 96% of staff witnessing and 84% admitting to such behavior within the past year. This study underscores that shortcomings in patient encounters remain a pressing issue, warranting further investigation. While this study provides qualitative insights, future research could adopt quantitative methods to examine the prevalence and specific impact of these shortcomings, using the findings here as a foundation.

To deepen our understanding, individual interviews with patients could capture more detailed accounts of poor treatment experiences and suggest targeted behavioral changes for staff. Additionally, future studies should aim to include more participants to ensure representation from less-visible patient groups and contexts, such as inpatient and outpatient care. One underexplored area in this study is the role of family members, which warrants further investigation. Participants’ insights suggested that family perspectives could provide valuable contributions to understanding and addressing shortcomings in psychiatric care.

## Conclusion

This study explored representatives’ views on shortcomings in patient encounters in Swedish adult psychiatry, identifying four themes: *Psychiatry is Inaccessible*, *Collaboration Does Not Occur*, *A Lack of a Holistic Perspective on the Person*, and *A Vulnerable Individual Meets a Powerful System*. While based on a small but diverse sample, the study indicates that concerns about poor treatment cut across diagnostic categories and geographic regions. These findings underscore the importance of reforms that strengthen respect, collaboration, and patient-centeredness in psychiatric care. Future research with larger and more varied samples is needed to further examine how systemic and interpersonal factors interact to shape patient experiences.

## Supplementary Information

Below is the link to the electronic supplementary material.

Supplementary Material 1

## Data Availability

The data that support the findings of this study are not publicly available in order to protect the integrity and anonymity of the research participants. Due to the sensitive nature of the information and ethical considerations, sharing of the raw data is not possible.
