# Supplementary material for: Patient and family organization perspectives on poor treatment in Swedish adult psychiatric care
Source: Discov Ment Health. 2026 Jan 22;6(1):16. doi: 10.1007/s44192-026-00372-0 (PMC12847503; doi:10.1007/s44192-026-00372-0)
Supplement: Supplementary file 1 — Supplementary Material 1 [file 44192_2026_372_MOESM1_ESM.docx]

**Supplementary material: Interview guide for the focus groups.**

1. As representatives of your organizations, what comes to mind when we say inadequate treatment?
   - What types of treatment deficiencies do your members encounter?
   - How common is it among your members?
   - (What recurring patterns can you identify in the stories?)
   - What risk factors contribute to experiencing inadequate treatment or to such deficiencies occurring?
     - Which groups are affected? (Diagnosis, gender, ethnicity, etc.)
     - Certain situations?
     - Certain types of care? (Inpatient, outpatient, emergency care, etc.)
     - Certain professional groups?
     - Other circumstances?
     - Is there anything you have noticed while listening to each other? Anything that stands out to you?
2. What do you perceive as the consequences of inadequate treatment?
   - (For the patients themselves?)
   - Are there individuals who are affected more severely or for whom the consequences are greater?
3. So far, we have focused on the patients themselves. What role do family members play when it comes to inadequate treatment in psychiatry? We are not referring to how family members treat patients, but rather how you perceive that family members are treated in their interactions with psychiatric services and how their involvement impacts the treatment that a patient receives in psychiatry.
   - How do you perceive that family members are treated in their interactions with psychiatry?
   - What are the consequences of this?
   - Does this, in turn, affect the patients? How?
   - How does the involvement of family members influence the treatment a patient receives in psychiatry?
   - In what ways? What are the consequences?

**Closing Questions**

- Is there anything you think we should have asked about? Or anything you were surprised that we did not ask about?
- If you could name one thing that you believe is needed to improve treatment in psychiatry, what would it be?
